# Kojic Acid Dipalmitate-Loaded Nanoparticles for the Treatment of Triple-Negative Breast Cancer

**Published:** 2026

**Authors:** Julia Capp Zilles, Onyinyechi Obidiro, Gantumur Battogtokh, Aline Rigon Zimmer, Renata Vidor Contri, Emmanuel O. Akala

**Affiliations:** 1 Center for Drug Research and Development, Department of Pharmaceutical Sciences, College of Pharmacy, Howard University, Washington, DC 20059, USA; 2 Programa de Pós-Graduação em Ciências Farmacêuticas–PPGCF, Universidade Federal do Rio Grande do Sul, Porto Alegre 90610-000, RS, Brazil

**Keywords:** Kojic acid dipalmitate, Polymeric nanoparticles, Dispersion polymerization, Cytotoxicity, Cellular uptake, TNBC

## Abstract

**Introduction:**

Triple-negative breast cancer (TNBC) is responsible for 10–20% of breast cancer cases and is associated with poor prognosis and limited treatment options due to the lack of expression of hormone and HER2 receptors. Kojic acid dipalmitate (KDP) is best known for its skin depigmenting activity; however kojic acid derivatives have shown promising antitumor activity. Such potential remains unexplored for KDP in breast cancer.

**Methods:**

KDP-loaded nanoparticles were developed by *in situ* dispersion polymerization, using polylactide (PLA: a biodegradable and biocompatible polyester) as a macromonomer and a pH-sensitive acetal crosslinker. The nanoparticles were characterized and tested in TNBC cells.

**Results and Discussion:**

The nanoparticles had a spherical morphology, a hydrodynamic diameter of approximately 240 nm with homogeneous size distribution, a negative zeta potential, and a drug loading of 0.61% ± 0.06 (w/w), in accordance with the drug loading theoretical value, with 100% encapsulation efficiency. FT-IR confirmed KDP incorporation into the nanoparticle. Nanoparticles were stable for 90 days (room temperature and 4°C storage). Cell viability studies with the TNBC cell line (MDA-MB-231) showed a significant, concentration-dependent reduction in cell viability following treatment with KDP-loaded nanoparticles, with an IC_50_ value of 2.04 μM at 48 hours, while blank nanoparticles were non-cytotoxic. *In vitro* cellular uptake studies with rhodamine 123–loaded nanoparticles demonstrated internalization of the nanoparticles after 1 hour, and a progressive accumulation in the perinuclear region up to 48 hours, consistent with the cytotoxicity plateau observed at longer exposure times.

**Conclusion:**

These findings highlight the novelty of pH-sensitive KDP-loaded polymeric nanoparticles and support suitability as a nanocarrier platform for poorly soluble drugs like KDP in triple-negative breast cancer research.

## Introduction

Breast cancer is the most commonly diagnosed type of cancer in women worldwide [1,2]. Different subtypes of breast cancer can be classified according to the expression of estrogen receptors (ER), progesterone receptors (PR), and human epidermal growth factor receptors (HER2) [3]. In clinical practice, they are classified into four different subtypes: luminal A (ER+/PR+, HER2−), luminal B (ER+/PR+, HER2±), HER2-enriched (HER2+), and triple-negative (ER−/PR−/HER2−), with each subtype guiding prognosis and therapy [4].

Triple-negative breast cancer (TNBC) lacks ER, PR and HER2 receptors, imposing challenges for its treatment as it does not respond to drugs that target those receptors [3]. This subtype of breast cancer accounts for 10–20% of the cases and is associated with poor outcomes [3,5–7], with five-year survival rates up to 16% lower than hormone receptor–positive breast cancers [5]. TNBC is also associated with breast cancer gene 1 (BRCA1) mutations, and tends to affect pre-menopausal women (under 40 years old), and disproportionally affects women of African ancestry [3,5].

Chemotherapy remains the primary treatment for TNBC, but resistance, relapse, and toxicity limit its effectiveness [3]. Surgery and radiotherapy are also being used [3]. More recently, immunotherapy, targeted therapy and combination therapy have also been explored [6], but drug resistance and cancer metastasis still remain. Nanotechnology-based therapies have the advantages of providing a controlled and targeted delivery of active substances, overcoming drug resistance and lowering side effects [7].

**Figure 1.**
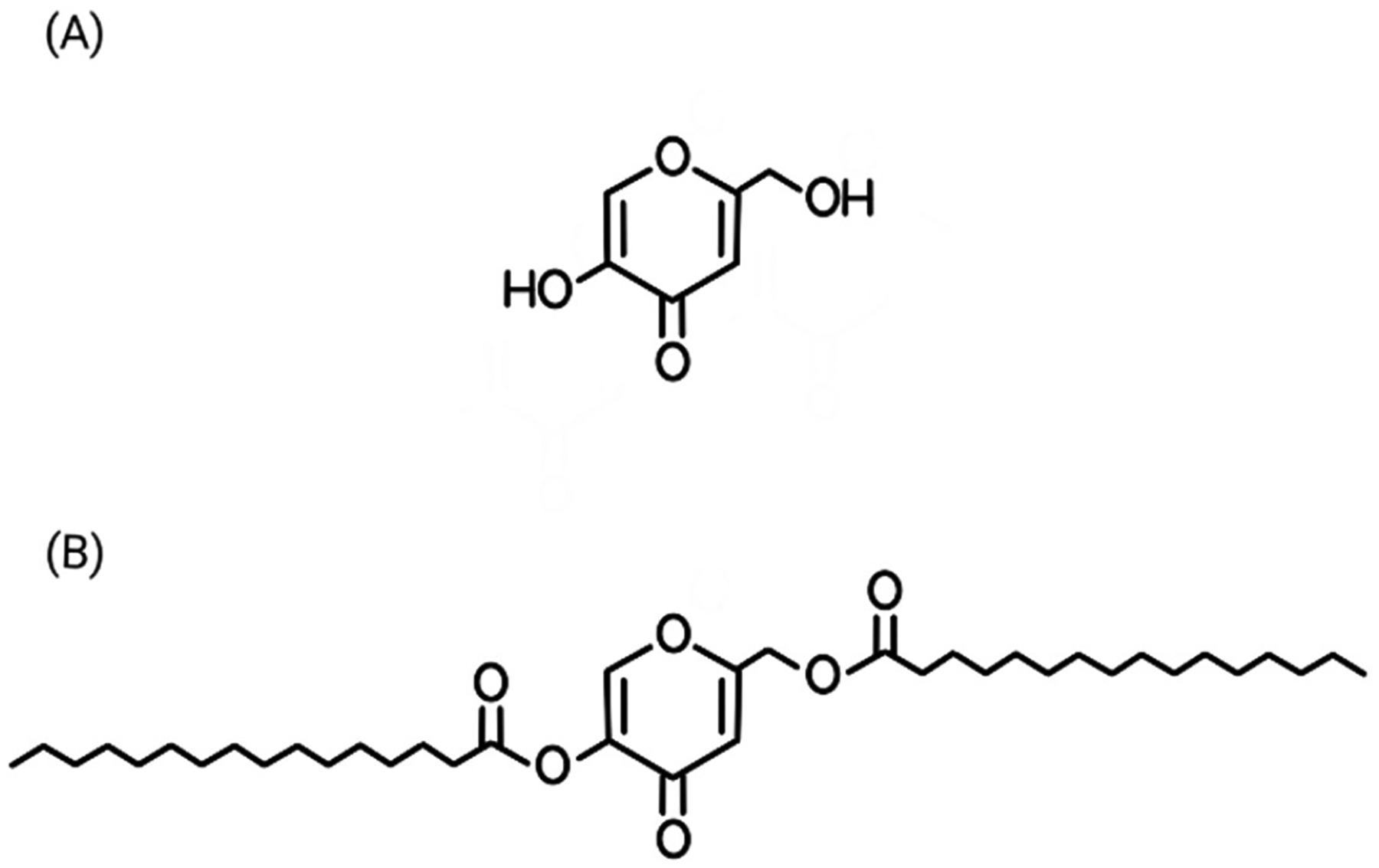
Chemical structure of kojic acid (**A**) and of kojic acid dipalmitate (**B**).

Kojic acid is an organic acid derived from fungi fermentation [8]. The most common use of kojic acid is as a skin depigmenting agent, inhibiting the key enzyme in melanin synthesis (tyrosinase). Besides that, antioxidant, antibacterial, anti-inflammatory, and antitumor activities have also been reported for kojic acid and its derivatives [9]. As kojic acid has low stability, being sensitive to light and heat [10], the use of derivatives such as kojic acid dipalmitate (KDP) comes as a more stable alternative to kojic acids use. KDP is the esterified form of kojic acid and it undergoes hydrolysis by esterases (esterases are enzymes that hydrolyze ester bonds, breaking them down into an acid and an alcohol), transforming it into kojic acid [11]. The chemical structure of KDP, showing esterification of kojic acid with two palmitic acid chains, is presented in Figure 1. Although KDP is a more stable option, it is a highly lipophilic compound, and strategies to facilitate its incorporation into formulations such as the use of nanotechnology are needed [12]. Hence, nanotechnology is considered appropriate to help solve this issue.

Besides facilitating incorporation of highly lipophilic drugs into formulations, nanotechnology-based approaches also have the advantages of providing a targeted delivery of active substances, controlling drug release, enhancing effectiveness at lower doses, hence lowering side effects [7]. Sustainable and environmentally-friendly approaches have been explored in the synthesis of nanomaterials for biomedical and therapeutic applications [13–15]. Recent advances in nanotechnology have reported green and simple synthetic approaches for functional nanomaterials with applications in analytical and biomedical contexts, including electrochemical sensing of bioactive compounds [16–18]. Among nanotechnological systems, polymeric ones stand out, as they can encapsulate both hydrophilic and hydrophobic drugs, protect the drugs from degradation and act as a drug reservoir, controlling the release at a targeted location [19]. Polymeric nanoparticles are suitable for the development of multifunctional nanoparticles [7]. For polymeric nanoparticles, obtaining them *via in-situ* dispersion polymerization technique offers advantages over the interfacial deposition of the preformed polymers technique, as the drug is present during the polymerization step. It allows the drug to be distributed within the polymer matrix, and can yield high encapsulation efficiency and prevent drug loss during manufacturing [20,21]. Further, in-situ dispersion polymerization techniques are suitable for thermolabile drugs because they can be carried out at ambient temperature by choosing appropriate initiators. Moreover, this one-pot synthesis can provide control over particle size and produce reproducible and monodisperse nanoparticles suitable for biomedical applications [20,21]. In the present work, KDP was incorporated into polymeric nanoparticles by physical encapsulation during *in situ* dispersion polymerization.

The current use of KDP is the topical treatment of skin hyperpigmentation. Besides the skin depigmenting properties, kojic acid derivatives also show promising antitumor potential [9], but it is still unexplored for KDP. Kojic acid derivatives have been studied in different cell lines, such as ovarian cancer [22], breast cancer [23], hepatocellular carcinoma [24], colon cancer [25] and melanoma [26,27]. None of them was associated with nanotechnology. For breast cancer, a lipophilic derivative of kojic acid (an allomaltol derivative, bearing 3,4-dichlorobenzyl piperazine moiety) is suggested to enter cancer cells through passive diffusion, showing cytotoxic effects in two different breast cancer cell lines: MCF7 and MDA-MB-231 [23]. MCF7 cells are luminal A breast cancer characterized by high estrogen receptor (ER) and progesterone receptor (PR) [4]. For this cell line, the lipophilic kojic acid derivative acted through apoptosis mechanisms, triggering caspase 8 and 9 activation, which led to overexpression of the pro-apoptotic gene p53 [23]. On the other hand, for MDA-MB-231, a TNBC cell line, the cell death was caused by necrosis due to reactive oxygen species (ROS) accumulation, as the compound enhanced LDH activity generating oxidative stress [23]. In this context, KDP is expected to be internalized by cancer cells, and may undergo intracellular hydrolysis by esterases, releasing kojic acid, and contributing to cytotoxicity in TNBC cells, likely through oxidative stress–mediated mechanisms.

The aim of this study was to develop pH-sensitive KDP-loaded nanoparticles using a one-pot *in situ* dispersion polymerization approach, and to characterize their physicochemical properties, stability and drug release behavior, as well as to investigate their cytotoxicity and cellular uptake in triple-negative breast cancer cells (MDA-MB-231). The present study incorporates KDP in a pH-sensitive biodegradable nanocarrier system to overcome limitations associated with formulation and delivery and explore its potential for triple-negative breast cancer therapy. To the best of our knowledge, kojic acid dipalmitate has not been incorporated into nanoparticle-based delivery systems for evaluation of its effects on triple-negative breast cancer, highlighting the novelty of the present nanotechnology-based approach.

## Materials and Methods

### Materials

Kojic acid dipalmitate (KDP) was purchased from SM Empreendimentos Farmacêuticos Ltda. (São Paulo, Brazil). Rhodamine 123 was obtained from Invitrogen (Waltham, MA, USA). Toluene (Chromasolv, HPLC grade, 99.9%), anhydrous dichloromethane (DCM, >99.8%), hexane, hydrochloric acid (ACS reagent, 37%), triethylamine (>99%), para-toluene sulfonic acid monohydrate (ACS reagent, >98.5%), tin(II) 2-ethylhexanoate (stannous octoate), 2-hydroxyethyl methacrylate (HEMA, 97%), 2,4-dimethoxybenzaldehyde (DMBA, 98%), sodium sulfate, phosphorus pentoxide, aluminum oxide and molecular sieves 4 Å (1.6 mm diameter) were purchased from Millipore Sigma (Burlington, MA, USA). Lactide and poly (ethylene glycol) n monomethyl ether methacrylate (PEG-MMA, n = 1000) were obtained from Polysciences (Warrington, PA, USA). Methanol and acetonitrile were obtained from Alfa Aesar (Ward Hill, MA, USA). Benzoyl peroxide (BPO) and N-phenyldiethanolamine (NPDEA) were purchased from Sigma-Aldrich (St. Louis, MO, USA). Ethyl acetate (ACS Reagent Plus, 99.8%), tetrahydrofuran (THF, HPLC grade), acetic acid and chloroform-d (99.8 atom% D) were obtained from Fisher Scientific (Waltham, MA, USA). Acetone was purchased from Oakwood Chemical (Estill, SC, USA). A 0.1 M sodium carbonate decahydrate (Na_2_CO_3_·10H_2_O) solution was prepared using the salt obtained from Acros Organics (Fair Lawn, NJ, USA).

### Methods

#### Synthesis and characterization of poly-L-lactide macromonomer

The macromonomer used for the development of the nanoparticles was synthesized according to methods previously reported [28,29], with modifications. L-(L)-lactide was recrystallized from toluene before use and the toluene was dried under vacuum. 2-Hydroxyethyl methacrylate (HEMA) was dried over activated molecular sieves and distilled under reduced pressure before use. Briefly, 12 g of lactide was polymerized in the presence of 1.8 mL of distilled HEMA, and 6 drops of stannous octoate by ring-opening polymerization. Prior to polymerization, the mixture was placed in a round-bottom flask put under vacuum for 10 minutes. The flask was then placed in an oil bath at 120°C with continuous stirring and nitrogen gas was flushed into it to create an inert atmosphere for 24 hours during polymerization. After that, the reaction was removed from the oil bath and allowed to cool. The obtained product was dissolved in 30 mL of dichloromethane, extracted 3 times with 50 mL of 0.1 M hydrochloric acid, and then washed 3 times with 50 mL of distilled water. The pure polymer was then precipitated with excess cold methanol, collected by filtration and dried in a vacuum oven over phosphorus pentoxide. A sample of the dried macromonomer was dissolved in chloroform-D for proton nuclear magnetic resonance (^1^H-NMR) (Bruker AVANCE 400 MHz NMR spectrophotometer) to verify its purity and determine the number average molecular weight. The molecular weight of synthesized macromonomer was analyzed by gel permeation chromatography (GPC) in the Reference Standard Laboratory (RSL) of the United States Pharmacopeia (USP) (Rockville, MD, USA). Fourier-transform infrared (FT-IR) spectroscopy was conducted using a PerkinElmer Spectrum 100 FT-IR spectrometer to further characterize the macromonomer.

#### Synthesis and characterization of acetal crosslinker

The pH-sensitive crosslinker agent was synthesized according to the procedure described by Berko and Akala (2020) [30]. 2-Hydroxyethyl methacrylate (HEMA) was dried over activated molecular sieves and distilled under reduced pressure before use. Activated molecular sieves, 19.9 g of 2,4-dimethoxybenzaldehyde (DMBA), 3.625 g of para-toluenesulfonic acid monohydrate, 60 mL of distilled HEMA, and 250 mL of anhydrous dichloromethane (DCM) were added to a round bottom flask with a magnetic stirrer inside. The reaction was stirred at room temperature for 30 minutes under nitrogen gas and then left to run for 24 hours. After that, the reaction was placed in an ice bath and 21 mL of triethylamine was injected into the flask to quench the reaction. It was stirred for 30 minutes at 0°C. The resulting product was filtered and washed with anhydrous DCM and rotary evaporated to give the liquid product.

The crude liquid product was then washed five times with 100 mL of a 0.1 M sodium carbonate decahydrate (Na_2_CO_3_·10H_2_O) solution using a separation funnel, and the organic phase was collected after each washing. Sodium sulfate was added to the organic layer to remove residual water, followed by solvent removal *via* rotary evaporation. The resulting crude product was purified by column chromatography using aluminum oxide as the stationary phase and a mobile phase composed of hexane/ethyl acetate (6:1, v/v) containing 1% trimethylamine. Samples were collected in test tubes, and thin-layer chromatography (TLC) was performed on all the samples to identify the fractions containing the crosslinker. After spotting each fraction, the TLC plates were visualized under UV light to detect the presence of the compound. Samples from fractions containing the product were prepared for proton nuclear magnetic resonance (^1^H-NMR). Pure fractions were combined and rotary evaporated [30].

A sample of the purified crosslinker was dissolved in chloroform-d and analyzed by ^1^H-NMR (Bruker AVANCE 400 MHz NMR spectrophotometer) to verify its purity. Liquid chromatography-mass spectrometry (LC-MS, Agilent 1260 Infinity system, Agilent Technologies, Palo Alto, CA, USA) was performed to verify the molecular weight. Fourier-transform infrared (FT-IR) spectroscopy was conducted using a PerkinElmer Spectrum 100 FT-IR spectrometer to further characterize the crosslinker.

#### Development of KDP-loaded polylactide nanoparticles

Kojic acid dipalmitate (KDP)-loaded nanoparticles were synthesized by one-pot dispersion polymerization using a redox initiator system composed of benzoyl peroxide/N-phenyldiethanolamine (BPO/NPDEA) as previously described [29,30]. Poly(ethylene glycol)n monomethyl ether methacrylate (PEG-MMA, n = 1000) was used both as a comonomer and a steric stabilizer. The poly(L-lactide) (PLA) macromonomer (0.240 mmol), crosslinker (1.136 mmol), PEG-MMA (0.504 mmol), and KDP (10 mg) were weighed separately into scintillation vials, dissolved in acetone, vortexed and combined. Water was then added to the mixture and the solution was transferred to a round bottom flask. Nitrogen gas was flushed into the reaction with continuous stirring at 100 rpm. NPDEA (0.196 mmol) and BPO (0.196 mmol) were injected into the reaction mixture at 10 min and 20 min, respectively, through a rubber closure. The nitrogen gas was stopped after 6h, and the polymerization was allowed to proceed for a total of 24 hours. The resulting nanoparticles were recovered by centrifugation. After centrifugation, the supernatant was collected and the nanoparticle pellet was redispersed in deionized water and lyophilized for 48 hours [29,30]. For the synthesis of fluorescent nanoparticles used in cellular uptake studies, KDP was replaced with 5 mg of rhodamine 123. Blank nanoparticles (without the drug) were also synthesized.

#### Characterization of the nanoparticles

##### Particle size and size distribution:

The average particle size and size distribution, and polydispersity index (PDI) were determined using the dynamic light scattering (DLS) technique. 5 mg of freeze-dried nanoparticles was suspended in 5 mL of deionized water, probe-sonicated for 5 minutes, and then filtered through a 0.45 μm syringe filter into a cuvette. The sample was then analyzed using a Brookhaven 90Plus particle size analyzer as previously described [29].

##### Zeta potential:

The zeta potential of the nanoparticles was analyzed by weighing 2 mg of freeze-dried nanoparticles and suspended it in 4 mL of previously filtered deionized water. The sample was thoroughly vortexed and then probe sonicated for 5 minutes and transferred to a cuvette for analysis using a Brookhaven 90Plus, ZetaPlus zeta potential analyzer as previously described [30].

##### Scanning Electron Microscopy (SEM):

The morphology of the nanoparticles was analyzed by scanning electron microscopy (SEM). The nanoparticles were diluted in deionized water, filtered using a 5 μm syringe filter. A drop of the sample was then placed on a carbon-coated stub and dried in a vacuum oven over a drying agent (phosphorus pentoxide) for 2 days, as previously described [29]. The sample was imaged using a HELIOS NanoLab 660 scanning electron microscope (Thermo Scientific, Hillsboro, OR, USA).

##### Drug loading:

The drug loading is the percent of drug (KDP) in the nanoparticle formulation. It was determined using a high-performance liquid chromatography coupled with ultraviolet detection (HPLC-UV) at 250 nm. Briefly, 2 mg of freeze-dried nanoparticles were dissolved in a solvent mixture of tetrahydrofuran (THF), acetonitrile, methanol, purified water, and acetic acid (35:30:29:5:1). The mixture was vortexed, probe-sonicated for 2 minutes and filtered through a 0.45 μm syringe filter into an HPLC vial. The analysis was performed according to Zilles *et al*. (2023) with modifications [12]. The sample was injected into a reverse-phase HPLC (RP-HPLC) system (Agilent-Hewlett Packard 1100 series) equipped with an Agilent Zorbax Eclipse Plus C18 column (150 × 4.6 mm, 5 μm) maintained at 25°C. An isocratic mobile phase consisting of THF, acetonitrile, methanol, purified water, and acetic acid (35:30:29:5:1) was used at a flow rate of 1 mL/min. Detection was performed at 250 nm. Prior to the sample analysis, a KDP calibration curve ranging from 1 to 100 μg/mL of KDP was performed in triplicate, and the linear regression equation of the average calibration curve was used to calculate KDP concentration in the samples. Data analysis was performed with ChemStation software (Agilent Technologies, Santa Clara, CA, USA).

Drug loading was calculated from this equation:

$$\%\text{DL}=\left(\left({\text{A}}_{\text{Sol}}\right)/\left({\text{A}}_{\text{Np}}\right)\right)\times 100\%$$

Where drug loading (DL) is the % weight of KDP in the freeze-dried nanoparticles. The weight of the freeze-dried nanoparticles is A_Np_ and amount of the loaded drug in the solution is A_sol_ which was analyzed using a reversed-phase high-performance liquid chromatography (RP-HPLC) method.

##### Encapsulation efficiency (EE):

Encapsulation efficiency is defined as the percentage of drug successfully encapsulated in the nanoparticles relative to the total amount of drug initially used in the formulation [30]. It was determined by quantifying the amount of free (non-encapsulated) drug present in the supernatant using HPLC-UV, with the method described in the previous section [12].

The amount of drug encapsulated was equal to the initial amount of drug used in the nanoparticle formulation during synthesis (A_prep_) minus the amount of drug found in the supernatant (A_sup_) after synthesis. Percent encapsulation efficiency was determined from the equation shown below:

$$\text{EE}\left(\%\right)=\left(\left({\text{A}}_{\text{Prep}}\right)-\left({\text{A}}_{\text{Sup}}\right)\right)/{\text{A}}_{\text{prep}})\times 100\%$$

##### FTIR of nanoparticles:

Fourier-transform infrared (FT-IR) spectroscopy was conducted using a PerkinElmer Spectrum 100 FT-IR spectrometer to further characterize the nanoparticles, as previously described [30]. Analyses were performed on KDP-loaded nanoparticles, blank nanoparticles and the drug.

##### *In vitro* drug release studies:

The *in vitro* drug release assay was conducted according to Adesina and coworkers (2014) with modifications [31]. A known amount of KDP-loaded nanoparticles was weighed, dispersed in 5 mL of release medium, and transferred to a 15 mL Falcon tube already containing 5 mL of release medium. The release medium consisted of 50% (v/v) 0.1 M acetate buffer (pH 5.0) with 0.1% of polysorbate 80 and 50% (v/v) THF. Because of the poor solubility of KDP, THF was added to the release medium. The tubes were then mounted on a Labquake^R^ shaker capable of 360° rotation and placed in an endotherm laboratory oven (Fisher Scientific, USA) set at 37°C. At designated time intervals, aliquots of the release medium were collected and replaced with an equal volume of fresh release medium to maintain constant volume conditions. Aliquots were analyzed by HPLC-UV as described above to quantify KDP release.

##### Stability assessment of nanoparticles:

The nanoparticles formulations were stored at room temperature (20°C) and under refrigeration (4°C) for 90 days. After the storage period, the nanoparticles were reanalyzed regarding particle size and size distribution, zeta potential and drug loading, following the methodologies described above.

#### Cell culture

##### Cell culture:

The human triple-negative breast cancer cell line MDA-MB-231 (ATCC HTB-26) was purchased from American Type Culture Collection (ATCC) (Manassas, VA, USA). Cells were cultured in L-15 medium, supplemented with 10% fetal bovine serum and 1% penicillin-streptomycin, and kept in an incubator at 37°C without CO_2_, according to the supplier’s recommendations (ATCC, HTB-26).

##### Cytotoxicity studies:

For the cytotoxicity assay, MDA-MB-231 cells were seeded at a density of 5 × 10^3^ cells per well in 96-well plates using 100 μL of complete L-15 medium and allowed to adhere overnight [7]. After 24 hours, the medium was replaced with 100 μL of treatment medium containing serial dilutions of KDP-loaded nanoparticles (NP-KDP), ranging from 1.25 to 20 μM. A separate group was treated with blank nanoparticles (without KDP) at a concentration corresponding to the amount of carrier used in the 20 μM NP-KDP condition. An untreated control group was also included. After incubation periods of 24, 48, and 96 hours, 100 μL of CellTiter-Glo reagent (Promega) was added directly to each well. The plate was shaken for 2 minutes and incubated at 37°C for additional 10 minutes. Luminescence was then recorded using a CLARIOstar microplate reader (BMG LABTECH) [7]. Experiments were performed in quadruplicate, and results were expressed as the mean ± standard deviation (SD) of these replicates.

##### Cellular uptake studies:

MDA-MB-231 cells were seeded at a density of 6 × 10^5^ cells per well in 6-well plates containing sterile glass coverslips using 2 mL of L-15 medium supplemented with 10% fetal bovine serum. After overnight incubation at 37°C to allow cell attachment, cells were treated with rhodamine 123-loaded nanoparticles, at a concentration of 2 μg/mL. Treatments were carried out for 1, 6, 24 and 48 hours at 37°C. Following incubation, cells were washed with room temperature PBS and stained with 1 mL of CellMask^™^ Deep Red plasma membrane stain for 10 minutes at 37°C. Cells were then washed again with warm PBS and fixed with 4% paraformaldehyde (1.5 mL) for 10 minutes at 37°C, followed by two washes with cold PBS. Nuclear staining was performed using Hoechst 33342 (1.2 μg/mL, 1.5 mL per well) for 10 minutes at room temperature. After a final cold PBS wash, coverslips were mounted onto glass slides with Fluoromount^™^ (Sigma-Aldrich, St. Louis, MO, USA) and dried overnight at room temperature [7,31]. Imaging was conducted using a spinning disk confocal fluorescent microscope. The system was a Yokogawa CSU X1 Spinning Disk Confocal with 405/488/561/640 lasers, an EMCCD camera (iXON), and a Nikon Ti-E PFS inverted scope microscope equipped with a 40× 1.49 NA TIRF-Apochromat oil immersion objective and a multitrack configuration. Fluorescence signals from Hoechst 33342, rhodamine 123-loaded nanoparticles, and CellMask^™^ Deep Red plasma membrane stain were collected using BP 405 nm, BP 488 nm, and BP 640 nm filters after excitation at 405, 488, and 640 nm laser lines, respectively. Images (512 × 512 pixels) were captured using the Nikon NIS imaging software.

#### Statistical analysis

All experiments were performed in triplicate unless otherwise stated, and results are presented as mean ± standard deviation (SD). Statistical differences among groups were evaluated using one-way analysis of variance (ANOVA), followed by Tukey’s post hoc test for multiple comparisons, using standard statistical procedures. Analyses were performed using GraphPad Prism^®^ version 8.0.2 (San Diego, CA, USA). A p-value < 0.05 was considered statistically significant.

## Results and Discussion

### Synthesis and characterization of poly-L-lactide (PLA) macromonomer

**Scheme 1.**
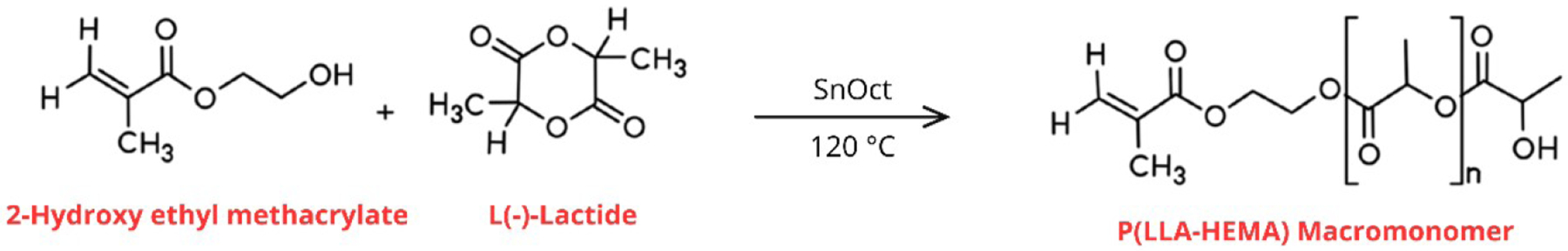
Synthesis of poly-L-lactide macromonomer.

The PLA-HEMA macromonomer was successfully synthesized by ring-opening polymerization [32]. L-Lactide was polymerized with HEMA in the presence of stannous octoate under nitrogen at 120°C (Scheme 1).

In order to confirm the formation of the PLA-HEMA macromonomer, ^1^H-NMR and FT-IR were employed. The ^1^H-NMR displayed signals at δ 5.6 ppm and 6.1 ppm, confirming the presence of the double bond (Supplementary Figure 1). The FT-IR spectrum (Supplementary Figure 2) displayed significant functional groups, such as C=O stretch at 1755.9 cm^−1^, corresponding to the HEMA vinyl functional group. Also, the spectrum displayed C-H bonds at 2997.7, 2946.6, 2873.7 cm^−1^ and C-O-C bonds at 1182.0, 1129.4, 1068.1, 1043.8 cm^−1^. These data confirm the formation of the PLA-HEMA macromonomer and are consistent with previous findings [28,29].

**Figure 2.**
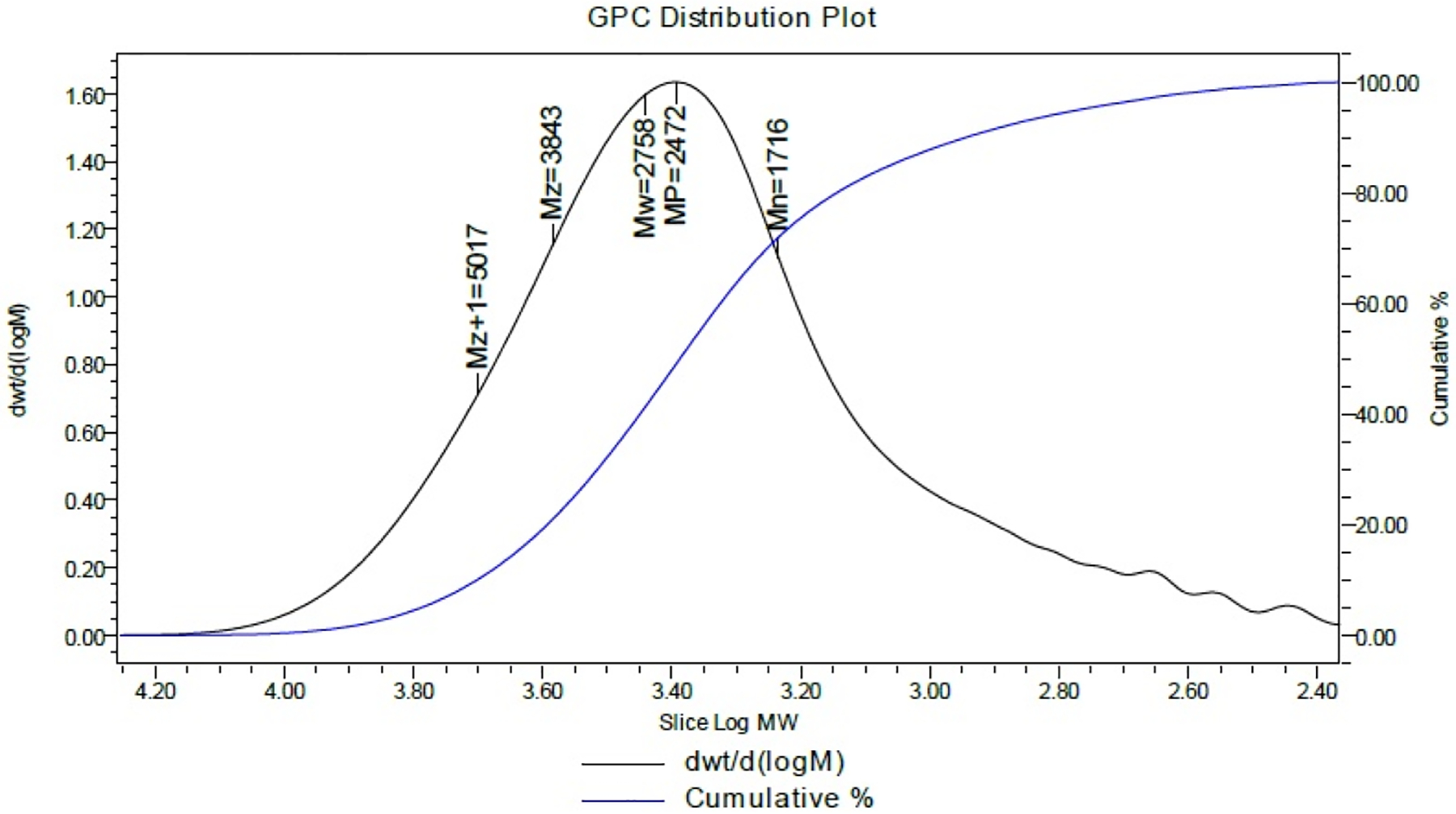
GPC of the HEMA-PLA macromonomer in THF.

The number-average molecular weight (Mn) of the PLA-HEMA macromonomer was determined by both ^1^H-NMR and GPC. The Mn was calculated to be approximately 2040 Da based on the ^1^H-NMR spectrum. The GPC analysis (Figure 2) revealed an Mn of 1716 Da, with a polydispersity index (PDI) of 1.61. This PDI reflects a moderately narrow distribution. The differences in the Mn values obtained from the ^1^H-NMR and GPC can be attributed to differences in the methodologies: the NMR provides a structure-based estimate, the GPC determines the molecular weight based on hydrodynamic volume [33]. Even though there is a slight variation, both methods indicate the formation of a low-to-moderate molecular weight polymer with acceptable dispersity for further application in nanoparticle synthesis.

### Synthesis and characterization of crosslinker

**Scheme 2.**
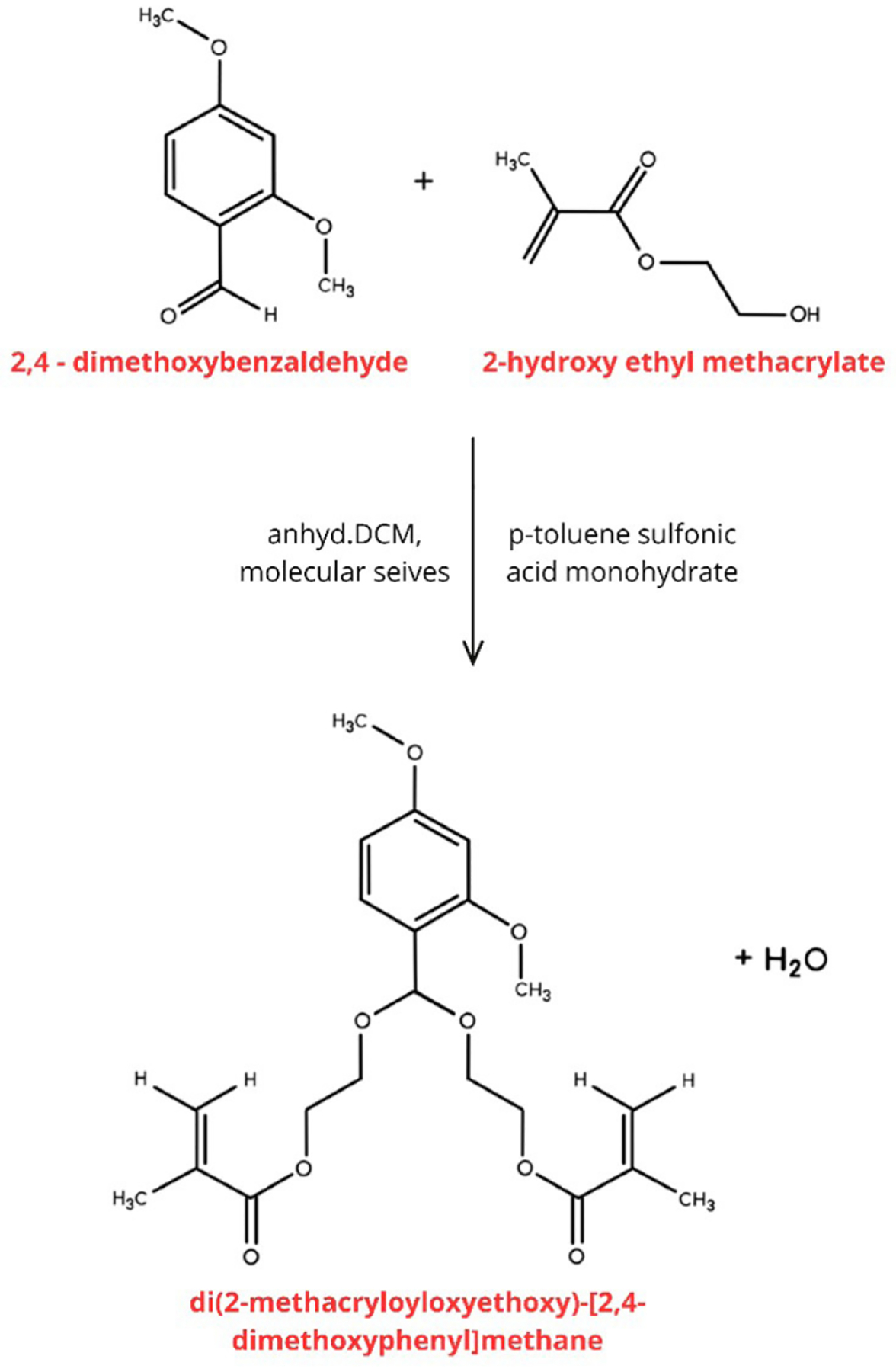
Synthesis reaction of the pH-sensitive acetal crosslinker.

The pH sensitive crosslinker was synthesized and purified according to previously reported methodology [30], and the reaction scheme is displayed in Scheme 2. Since tumors have an acidic environment due to lactate secretion from anaerobic glycolysis, which correlates with tumor progression and poor prognosis [34], incorporating a pH-sensitive crosslinker into nanoparticle matrix can lead to a targeted and rapid delivery of the drug in the acidic environment [35].

**Figure 3.**
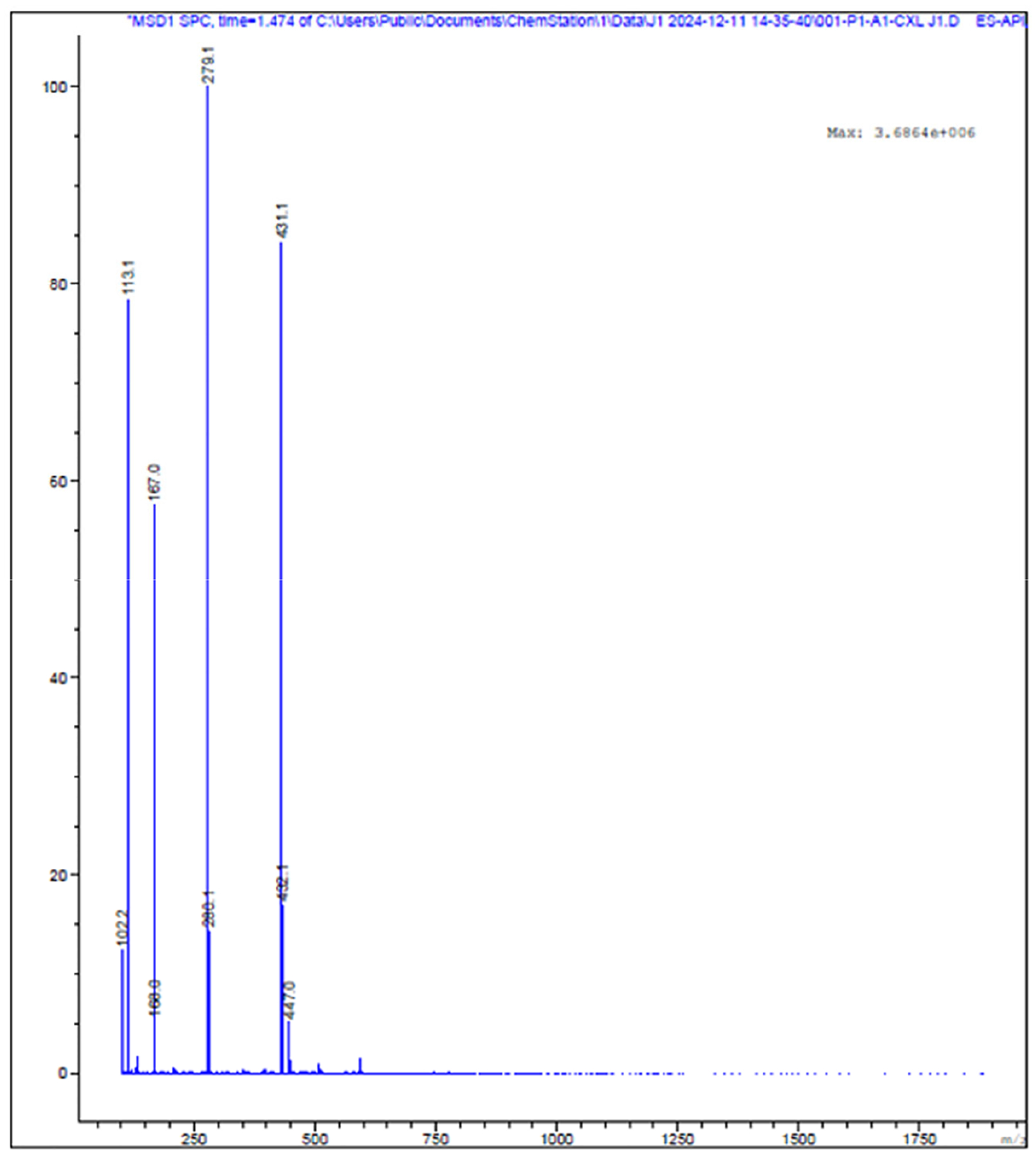
LC-MS spectrum of the purified crosslinker.

The ^1^H-NMR displayed the acetal peak signal at around δ 5.8 ppm, which confirmed the formation of the pH-sensitive acetal crosslinker (Supplementary Figure 3). Moreover, there were no signals at around δ 10.5 ppm, further confirming the absence of the aldehyde peak from the reaction precursor, indicating purity of the product. The FT-IR spectrum (Supplementary Figure 4) showed significant functional groups, such as C=O stretch from ester carbonyl groups from the methacrylate moieties at 1715.4 cm^−1^, C-H stretch from ethoxy side chains and methacrylate backbone at 2957.8, 2929, 2876, 2838.3 cm^−1^, and C-O and C-O-C typical of ester bonds and ether linkages at 1295.29 – 1002.15 cm^−1^. According to the molecular formula, the molecular weight of the crosslinker should be 408 g/mol. LC-MS analysis was used to determine the molecular weight. The observed peak at 431.1 (Figure 3) confirmed the expected molecular weight (408 g/mol + 23 g/mol from Na^+^). The sodium atom (23 g/mol) could have appeared due to the washes with sodium carbonate decahydrate. The result is in accordance with previous findings from Berko and Akala (2020) [30].

### Development and characterization of KDP-loaded polylactide nanoparticles

Following the synthesis of the PLA macromonomer and of the pH-sensitive acetal crosslinker, the KDP-loaded nanoparticles were synthesized by the *in situ* dispersion polymerization method. The one-pot synthesis was followed by freeze-drying of the nanoparticles and led to a homogeneous powder.

**Figure 4.**
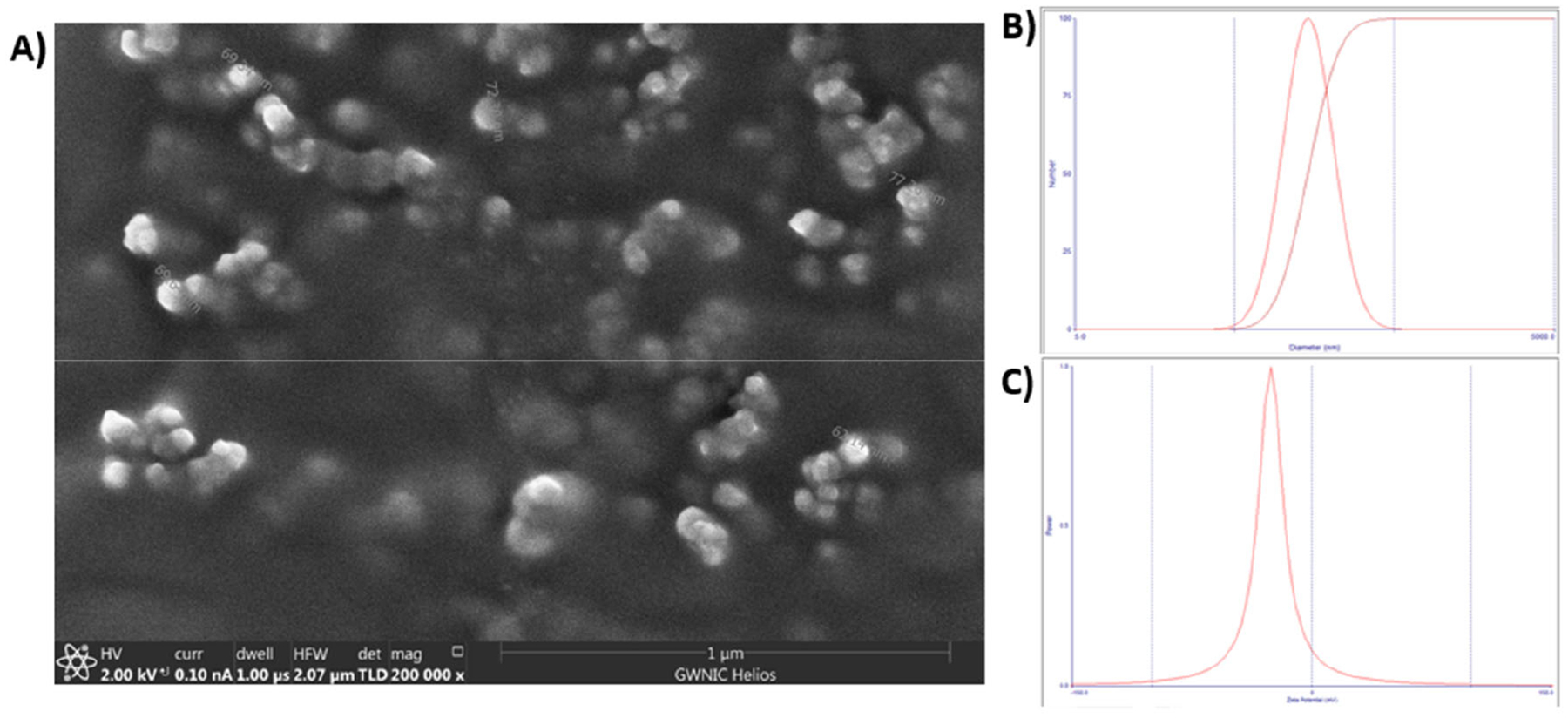
(**A**) Typical SEM image of the KDP-loaded nanoparticles, (**B**) particle size and polydispersity index of KDP-loaded nanoparticles and (**C**) Zeta potential of KDP-loaded nanoparticles.

Scanning electron microscopy (SEM) showed a spherical morphology, as depicted in Figure 4A, with a particle diameter of around 70 nanometers. Table 1 shows the average particle size and size distribution obtained by the dynamic light scattering (DLS) technique, zeta potential, drug loading, and encapsulation efficiency of the KDP-loaded nanoparticles and of blank nanoparticles. The incorporation of KDP into the nanoparticles did not interfere with the nanometric features. The mean diameter obtained by DLS was higher than the one obtained by SEM, which could be attributed to the differences between the two techniques. While SEM measures the dry-state nanoparticle size, DLS measures the hydrodynamic diameter of the nanoparticles in dispersion, capturing solvation layers, which could lead to larger size values [36]. Nevertheless, both analytical techniques revealed an appropriate particle size for polymeric nanoparticles suitable for drug delivery [7,30,37,38]. The DLS technique also showed a monomodal size distribution (Figure 4B) with a polydispersity index (PDI) below 0.2. Although nanoparticles in the 100–200 nm range are often considered optimal for tumor accumulation *via* the enhanced permeability and retention (EPR) effect, several studies have shown that polymeric nanoparticles with sizes up to ~300 nm can still accumulate in tumors depending on tumor type, vascular permeability, and nanoparticle surface properties [7,31,38]. The zeta potential analysis (Figure 4C) indicated a negative surface charge, and since it had an absolute value higher than 25 mV, the electrostatic contribution is the main mechanism of stabilization of the nanoparticle [39].

Drug loading and encapsulation efficiency (EE) were determined by HPLC-UV. Prior to the sample analysis, three KDP calibration curves with concentrations ranging from 1 to 100 μg/mL of KDP were performed, and the linear regression equation of the average calibration curve was used to calculate KDP concentration in the samples. A correlation coefficient of 0.9993 was obtained (data not shown), showing that the method was linear over the range of 1–100 μg/mL, and also specificity was confirmed. The theoretical value for drug loading was 0.62%, and the obtained value was 0. 61% ± 0.06, indicating that there were no losses during the manufacturing process. This result is consistent with the advantages of the *in-situ* dispersion polymerization technique, where drug incorporation occurs with the polymer formation, yielding complete (or near complete) recoveries of drug loading [20]. Furthermore, the highly lipophilic nature of KDP contributed to its favorable encapsulation in the lipophilic cores of the nanoparticles. The encapsulation efficiency (EE) was measured by quantifying the amount of KDP in the supernatant after centrifugation and prior to freeze-drying [30]. The EE was calculated as 100%, as no KDP was detected in the supernatant, further highlighting the efficiency of this manufacturing technique.

**Figure 5.**
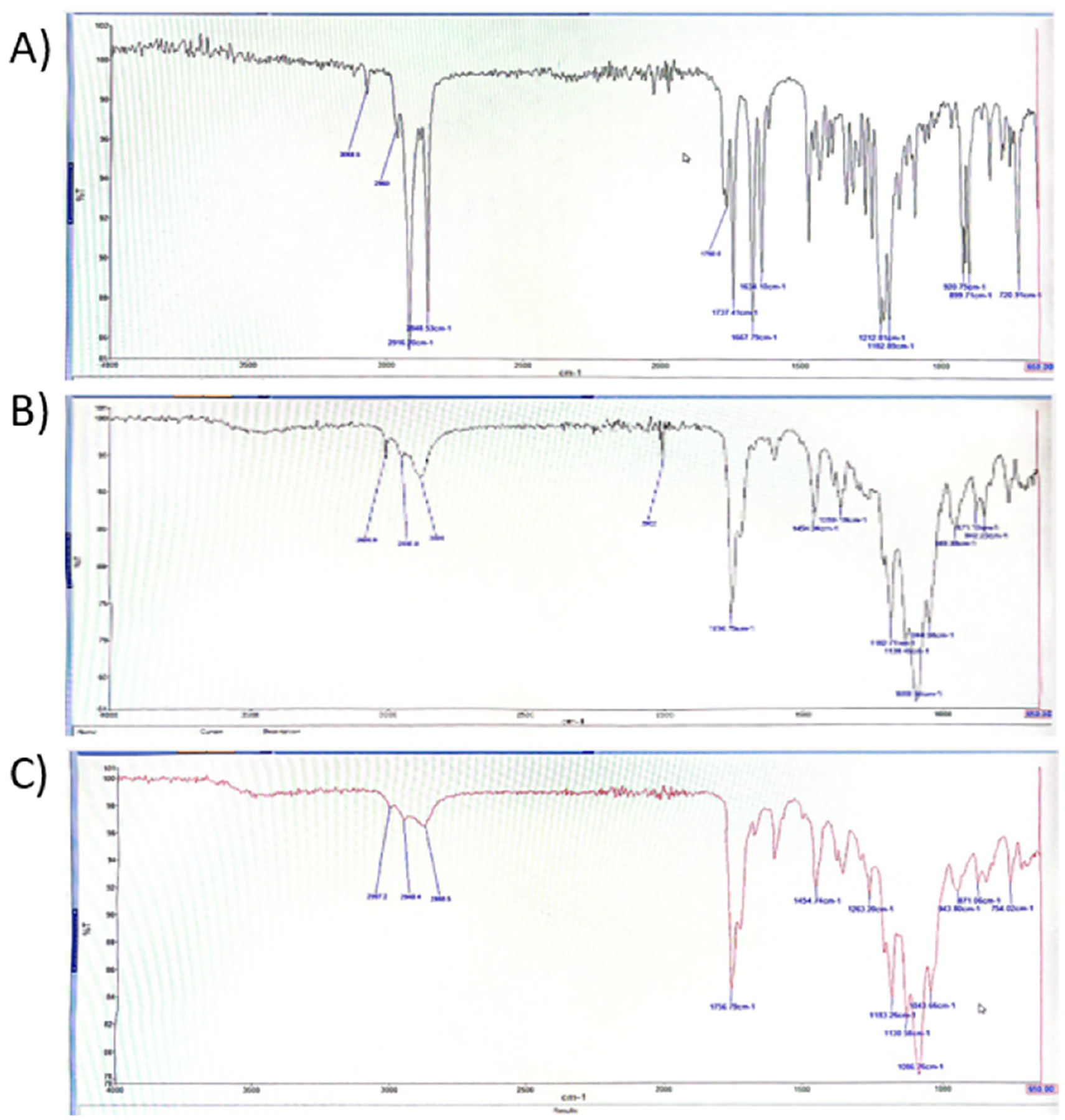
FTIR spectrum of KDP (**A**), the blank nanoparticle (**B**) and KDP-loaded nanoparticle (**C**).

The FT-IR spectra confirmed the successful incorporation of KDP into the polymeric nanoparticles (Figure 5). It can be observed that KDP-loaded nanoparticles retained the main polymer absorption bands. It also showed overlapping bands with KDP, especially in the C-O region (1250–1050 cm^−1^). Moreover, the absence or reduction of drug-specific peaks in the nanoparticle spectrum indicates that the drug is encapsulated without altering the structure of the nanoparticle [40,41].

#### Drug release

**Figure 6.**
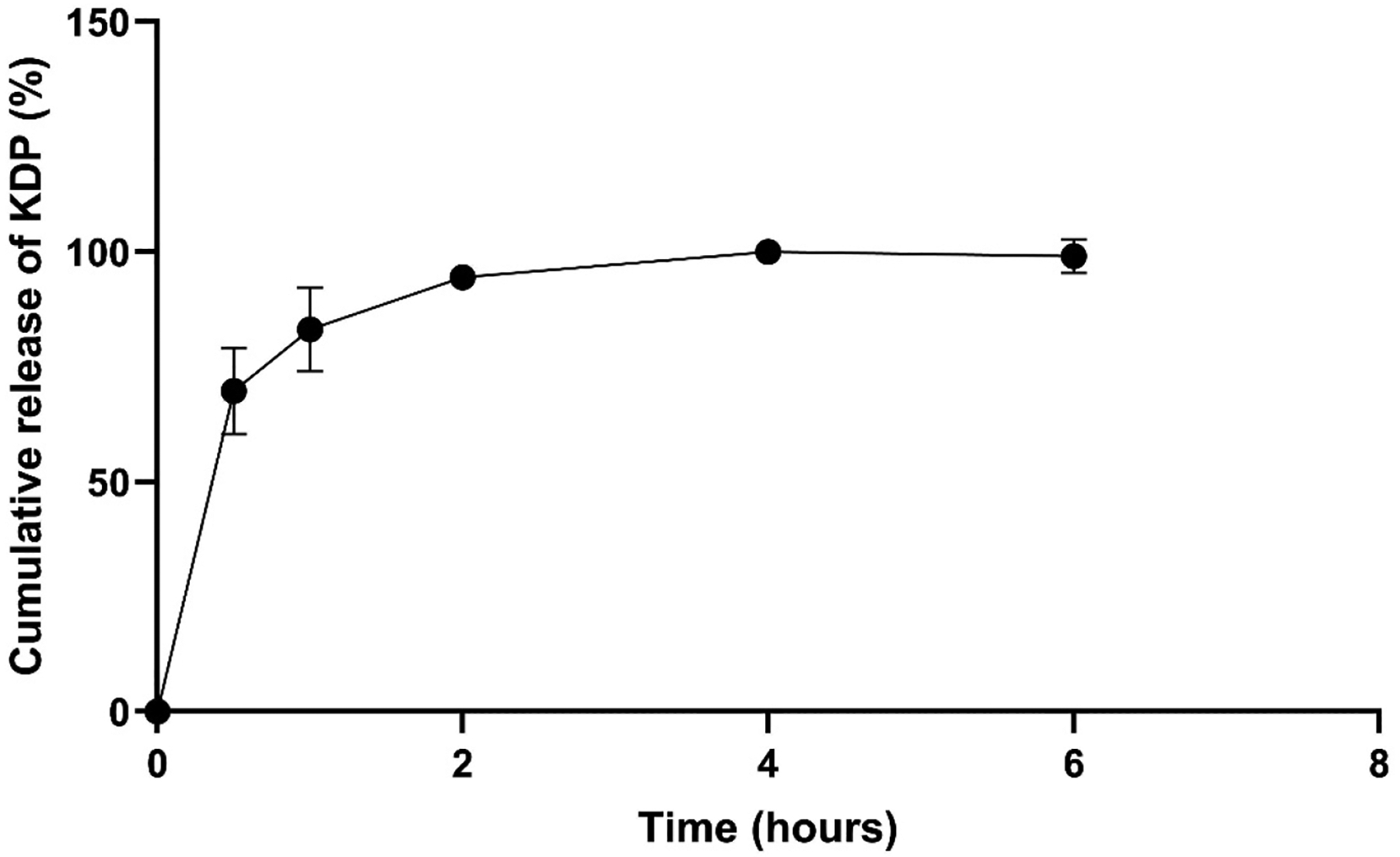
*In vitro* drug release study. Cumulative release profile of KDP from KDP-loaded nanoparticles at pH 5.0.

An *in vitro* drug release study was conducted using a release medium composed of 50% acetate buffer (pH 5.0, containing 0.1% polysorbate 80) and 50% THF. The release profile was fast, with a rapid initial burst, followed by a sustained release that reached a plateau (Figure 6). Around 70% of KDP was released in the first 30 minutes, and a cumulative release of 100% was reached after 4 hours of experiment, after which the curve plateaued. The initial burst release is probably due to KDP adsorbed on or near the nanoparticle surface, while the subsequent slower release reflects diffusion from the polymer matrix.

THF was included in the release medium in order to guarantee the solubilization of the released KDP and allow for its quantification. However, due to its presence, it probably interfered with the nanoparticle’s structure by disrupting the polymeric matrix, accelerating the release of KDP. The observed drug release was probably faster than would be expected under physiological conditions. Importantly, 50% of the release media was acetate buffer at pH 5.0. This pH mimics the acidic environment of tumor sites [34], adding biological relevance to assay conditions, and suggesting that similar burst release could occur intracellularly, where endosomal/lysosomal compartments are acidic. Although the release is faster than would be expected under purely physiological conditions, the study confirms successful encapsulation and release potential of KDP.

Interestingly, despite the presence of the THF, the release profile of the drug still exhibited a certain degree of control, with complete KDP release occurring only after several hours. A fast release at acidic pH, such as the one found in tumor microenvironment, can enable fast intracellular drug availability. Previous studies from our group using the same pH-sensitive acetal crosslinker demonstrated accelerated drug release at acidic pH (5.0) compared to neutral pH (7.4), confirming the pH-responsive behavior of this system [35,38].

Smart polymeric systems have been widely explored to achieve high loading capacity and controlled and sustained release through rational material design and structural tunability [42,43]. These studies highlight how polymer architecture and assembly conditions play a key role in loading efficiency and release behavior, supporting the design strategy adopted in the present work.

The use of organic solvents in drug release studies with poorly soluble drugs has already been reported. Adesina and coworkers (2014) employed octanol, an organic solvent, as a release medium for paclitaxel-loaded nanoparticles [31]. The authors observed a biphasic release profile, with a burst release in the first hours, which was then slowly increasing and reaching a plateau after several days [31]. In contrast, in the present study, we observed a rapid burst release that quickly reached a complete release and plateaued, suggesting that part of the KDP may be adsorbed on the nanoparticle surface. Moreover, the presence of THF likely disrupted the nanoparticle structure, contributing to an accelerated release profile. While the data obtained from the drug release studies cannot be correlated with biological data because of the high concentration of THF, the data further demonstrated the encapsulation of KDP.

#### Stability

The three batches of the KDP-loaded nanoparticles were stored at room temperature and at refrigerated storage condition (4°C) for 90 days and analyzed. No statistically significant differences were observed (p>0.05) regarding particle size, zeta potential, and drug loading (Supplementary Figure S5), demonstrating that the freeze-dried nanoparticles were stable regardless of the temperature of storage. Zilles and coworkers (2023) developed KDP nanoemulsions that were analyzed in terms of stability after 30 days of storage [12]. Although they also reported stable nanofeatures, the drug content at 25°C suffered a 30% decay, which was attributed to hydrolysis of the drug in the aqueous nanoemulsion medium [12]. Indeed, lyophilization of nanoparticles, as carried out and observed in this work, can lead to the achievement of long-term stability, preserving both nanofeatures and drug content [44]. Therefore, lyophilization can prevent ester-containing drugs like KDP from hydrolysis, enhancing its stability.

### Cell culture

#### Cytotoxicity studies

**Figure 7.**
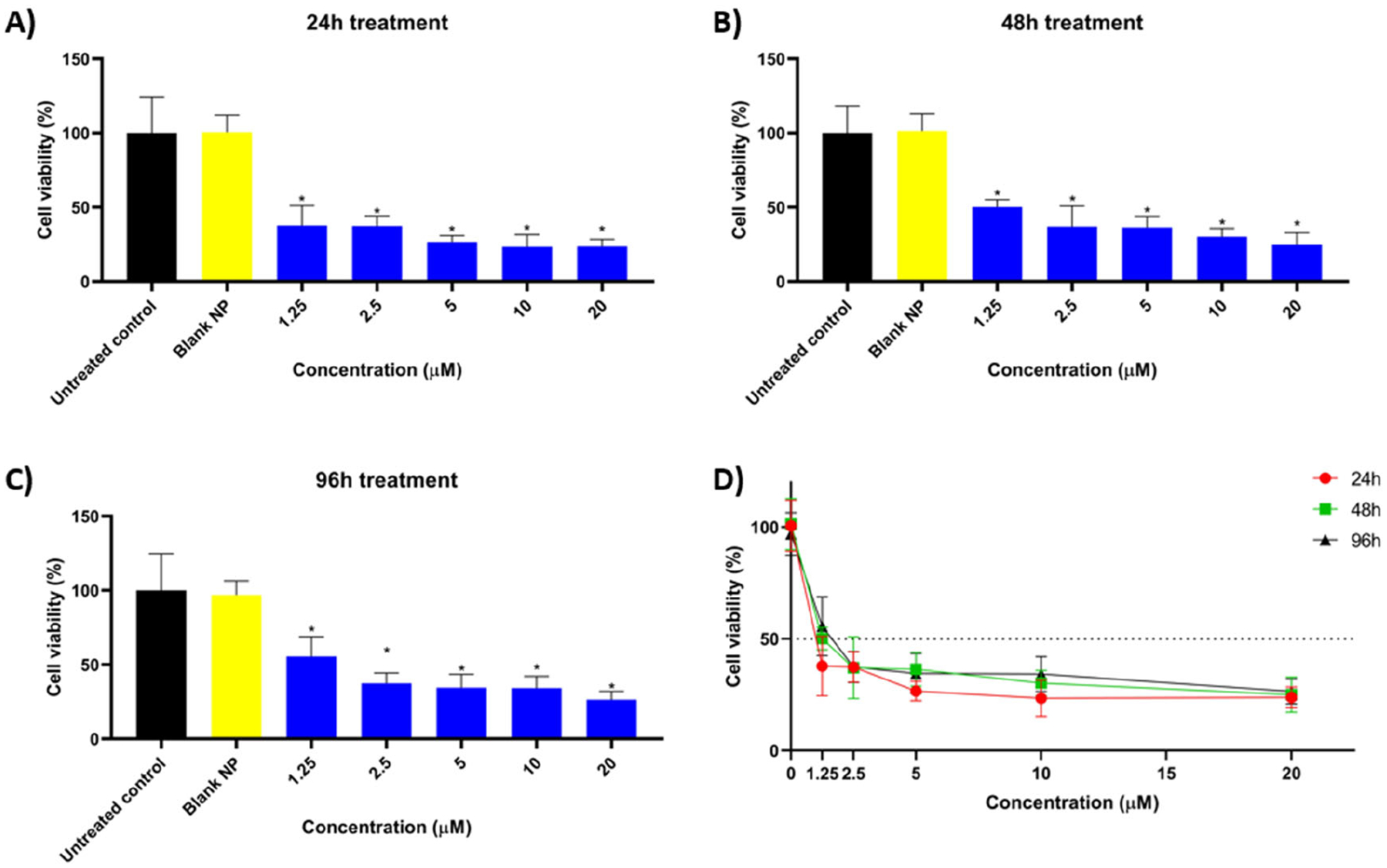
Cytotoxicity study by CellTiterGlo^®^ luminescence assay. Effect of treatment with blank nanoparticles (yellow) or KDP-loaded nanoparticles (blue) on MDA-MB-231 cells after 24 hours (**A**), 48 hours (**B**) and 96 hours (**C**). Effect of treatment duration with KDP-loaded nanoparticles on cytotoxicity on MDA-MB-231 cells.

In order to analyze the cytotoxicity of KDP-loaded nanoparticles, the Cell TiterGlo^®^ assay was employed. This assay measures the cell viability based on ATP luminescence, directly reflecting metabolically active cell number [7,45]. The formulations were used to treat human triple-negative breast cancer (TNBC) cell line MDA-MB-231. TNBC cell line lacks ER, PR and HER2 receptors [3]. Untreated cells were used as control. Figure 7 displays the percent cell viability of the cells after 24h (**A**), 48h (**B**) and 96h (**C**), as well as a comparison of the effect of treatment duration (**D**). It was found that treatment with KDP-loaded nanoparticles led to a statistically significant (p<0.05) decay in cell viability in comparison to the untreated control even from the lowest concentration (1.25 μM), at all time points. Moreover, at all time points, the cell death was slightly higher with increasing concentrations of the formulation (p>0.05).

Ercan and coworkers (2020) tested several allomaltol (kojic acid analogue) derivatives and obtained decays in cell viability of MDA-MB-231 after 48-hour treatment using the MTT assay [23]. The MTT assay investigates cell viability based on mitochondrial activity, and it is based on the NADH-dependent reduction of yellow tetrazolium salt (3-(4,5-Dimethylthiazol-2-yl)-2,5-diphenyltetrazolium bromide) (MTT) to purple formazan crystals via colorimetric readout [46]. The reduction in cell viability in the study of Ercan and coworkers (2020) was attributed mainly to necrosis linked to reactive oxygen species accumulation and membrane damage, with limited induction of drug resistance [23]. The author obtained an IC_50_ value of 47.8 μM for the derivative tested. In the present investigation, decays in cell viability were observed, with the KDP-loaded nanoparticles resulting in an IC_50_ value of 2.04 μM after 48-hour treatment. The lower IC_50_ value of KDP compared to the kojic acid derivative tested by Ercan and coworkers (2020) could be related to differences among the compounds, as structural modifications of kojic acid analogues can alter their biological activity, and also due to the contribution of nanotechnology. Nanotechnology can enhance the activity of a drug and lead to a targeted delivery on the cancer site, and also improve the delivery of poor water-soluble drugs, such as KDP [3].

The IC_50_ value of 2.04 μM after 48-hour treatment obtained with the KDP-loaded nanoparticles treatment indicates a considerable cytotoxicity against MDA-MB-231 cells. Gantumur and Akala (2025) tested paclitaxel and cisplatin-loaded nanoparticles, functionalized or not with cetuximab, in the same cell line and using the same assay (CellTiterGlo^®^). Paclitaxel and cisplatin are drugs widely employed in TNBC treatment that when in combination can have synergistic effects [7]. The authors reported, after 96-hour treatment, an IC_50_ of 0.57 μM for the free drug combination (paclitaxel and cisplatin), 0.49 μM for the drug-loaded nanoparticles, and an even lower IC_50_ value (0.10 μM) was obtained for the functionalized nanoparticle [7]. This result showed that the nanotechnological approach enhanced cytotoxicity, and functionalizing the nanoparticles can further potentiate its therapeutic effect.

To the best of our knowledge, there are no other studies with KDP and TNBC cells, highlighting a possible new drug for this challenging breast cancer subtype. Moreover, Zilles and coworkers tested KDP nanoemulsions on murine fibroblasts (3T3-L1 cells), which are a non-cancerous cell line, and observed no cytotoxic effects at a maximum concentration of 16.15 μM of KDP (1% of 1 mg/mL KDP nanoemulsion) [12]. Zilles and coworkers also tested KDP-loaded nanocapsules in fibroblasts (3T3 cells) and normal human epidermal melanocytes (NHEM) and the results also indicated acceptable biocompatibility [47].

Longer treatment durations with KDP-loaded nanoparticles did not lead to higher cell death in MDA-MB-231 cells (Figure 7D). This could be explained by a saturation in nanoparticle internalization, limiting intracellular delivery [48]. This result also aligns with the results from Puri and coworkers (2018), who reported that nanoparticle cytotoxicity depended mainly on concentration rather than prolonged exposure in prostate cancer cell lines [38], although Adesina and coworkers (2014) observed, in MDA-MB-231 cells, increased effects of the nanoparticle treatment up to 96h that plateaued at higher doses [31].

Nanoparticles should be biocompatible and biodegradable to be used as drug carriers [49]. The polylactide (PLA) nanoparticles investigated in this work are biodegradable and biocompatible (PLA is approved by the US FDA for use in humans). Belonging to the family of polyesters, PLA is known to exhibit adequate biodegradability and biocompatibilty. Under physiological conditions, polyesters are generally degraded by hydrolysis into products which are well tolerated by various tissues. For example, the degradation products from PLA, PGA and PLGA, namely glycolic acid and lactic acid, are physiological substances easily eliminated through the Krebs cycle. The KDP-loaded nanoparticles are likely internalized *via* endocytosis, where the intracellular environment, particularly acidic endosomal and lysosomal compartments, can facilitate the hydrolytic degradation of PLA and cleavage of the pH-sensitive acetal crosslinker. This degradation leads to the controlled release of KDP directly within the cells, contributing to the observed cytotoxic effects.

The blank nanoparticles investigated in this work were also tested and showed no effects on cell viability regardless of the duration of the treatment (Figure 7). Hence, the cytotoxicity of the KDP-loaded nanoparticles can be attributed to the presence of KDP in the nanoparticles. From a biological perspective, nanoparticle-based delivery systems are advantageous for cancer therapy as they can enhance drug stability, improve cellular interaction, and promote intracellular delivery, increasing therapeutic efficacy while reducing adverse effects. These features support the use of pH-sensitive nanoparticles as a rational strategy to enhance the antitumor potential of KDP in triple-negative breast cancer cells [50,51].

#### Cellular uptake studies

**Figure 8.**
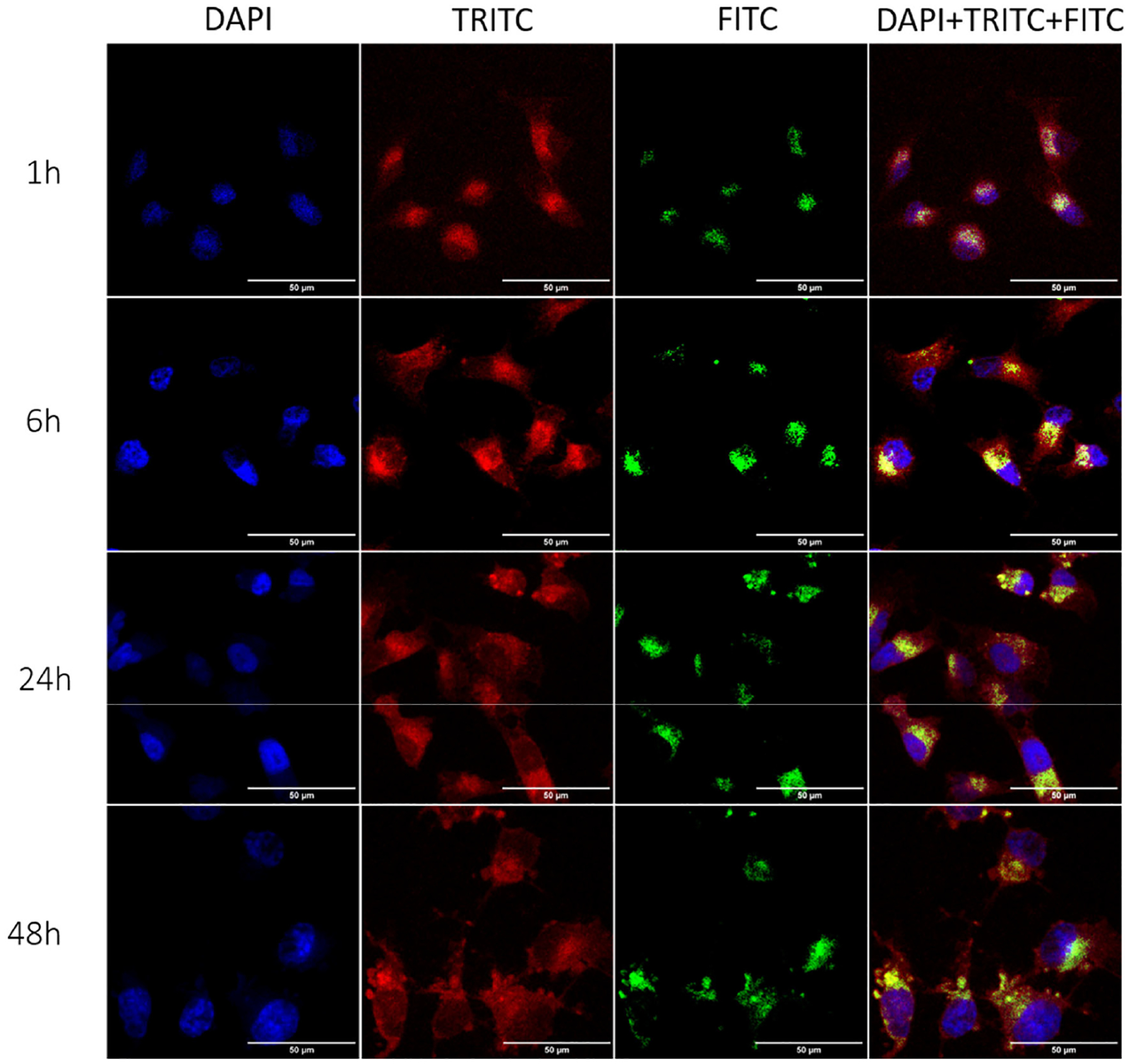
Cellular uptake of rhodamine 123-loaded nanoparticles by confocal microscopy after 1, 6, 24 and 48 hours of incubation in MDA-MB-231 cells. DAPI channel (Hoechst, blue) indicates the nucleus, TRITC channel (CellMask Red, red) indicates the cell membrane, and FITC channel visualizes rhodamine 123 (green).

The cellular uptake of rhodamine 123–loaded nanoparticles was evaluated in MDA-MB-231 cells after 1, 6, 24, and 48 hours of incubation. These nanoparticles were prepared the same way as the KDP-loaded ones, but with rhodamine 123, instead of KDP, due to its fluorescence. The cell nucleus was stained with Hoechst 33342 (detected in the DAPI channel, blue), the plasma membrane with CellMask^™^ Deep Red (detected in the TRITC channel, red), and nanoparticles were visualized through rhodamine 123 fluorescence (detected in the FITC channel, green). Figure 8 shows each of the channels separately and also the merged image at each of the time points. Fluorescence signals indicated that nanoparticles were already internalized by the cells after 1 hour of incubation. At later time points (6, 24 and 48 hours), intracellular localization became progressively more concentrated in the perinuclear region, suggesting transport of nanoparticles closer to the nucleus.

The cellular uptake assay was in accordance with the cytotoxicity findings, showing that nanoparticles were already internalized by MDA-MB-231 cells after 1 hour of treatment and accumulated in the perinuclear region over time, consistent with the observed plateau in cell death despite prolonged exposure. Similar results were found with PLA nanoparticles loaded with paclitaxel in MCF7 cell line (luminal A breast cancer cell line), where internalization was observed within 1 hour [31]. Similarly to the nanoparticles in the present study, Adesina and coworkers (2014) developed nanoparticles (also prepared by dispersion polymerization) using PLA as a macromonomer and encapsulating a lipophilic drug [31], and the data showed that polylactide nanoparticle is a good carrier option to target cancer cells. More recently, Gantumur and coworkers (2025) developed dual-loaded polymeric nanoparticles, with paclitaxel (lipophilic) and cisplatin (hydrophilic), and attached cetuximab (monoclonal antibody) to them to enhance targetability and reported that cellular uptake of the nanoparticles was enhanced in a time-dependent manner in MDA-MB-231 cells, from 6 to 48 hours [7]. Compared with these studies, our findings further support that polymeric nanoparticles are rapidly internalized by TNBC cells and accumulate in the perinuclear region. Moreover, we highlight the compatibility of PLA-nanoparticles as a system for the delivery of lipophilic drugs in cancer therapy.

## Conclusion

In summary, both the PLA macromonomer and the pH-sensitive crosslinker were successfully synthesized and characterized, confirming the formation of pure products. The *in situ* dispersion polymerization method led to the formation of KDP-loaded nanoparticles with a drug-loading of 0.61% ± 0.06, virtually the same as the theoretical value based on the quantity of drug added during the fabrication of the nanoparticles. The nanoparticles had spherical morphology, a hydrodynamic diameter of approximately 240 nm with homogeneous size distribution, and a negative zeta potential. The drug was successfully encapsulated within the nanoparticles. In biological assays using triple-negative breast cancer cell line MDA-MB-231, KDP-loaded nanoparticles significantly reduced the cell viability (p < 0.05) from the lowest concentration tested (1.25 μM), while blank nanoparticles showed no cytotoxicity, confirming their biocompatibility. Duration of the treatment did not affect the cytotoxic response significantly, consistent with the rapid cellular uptake and accumulation of nanoparticles close to the cell nucleus. Taken together, the results highlight the novelty of the pH-sensitive KDP-loaded nanoparticles and of their potential as a promising candidate for further studies in TNBC therapy.

From a practical perspective, this nanoparticle system may serve as a platform for the delivery of highly lipophilic drugs, particularly in tumor microenvironments characterized by acidic pH. The one-pot *in situ* dispersion polymerization approach also offers a reproducible and adaptable strategy for developing pH-sensitive nanocarriers, overcoming limitations related to techniques such as nanoprecipitation. Despite the encouraging findings, the study has limitations related to the lack of *in vivo* animal investigations, that should be addressed before clinical translation can be considered. Challenges for translation include validation of *in vivo* efficacy and safety, as well as assessment of scalability and reproducibility of the nanoparticle system. Thus, future studies are needed to better understand the therapeutic potential of these KDP-loaded nanoparticles in TNBC, including *in vivo* evaluation, biodistribution, pharmacokinetics, and safety.

## Supplementary Material

Supplementary Material

## Figures and Tables

**Table 1.** Characterization of KDP-loaded nanoparticles.

	KDP-loaded nanoparticles	Blank nanoparticles
**Particle size (nm)**	241.07 ± 36.65	267.67
**PDI**	0.164 ± 0.011	0.158
**Zeta potential (mV)**	−46.92 ± 5.67	−43.57
**Drug loading (%, w/w)**	0.61 ± 0.06	-
**Encapsulation efficiency (%)**	100%	-
