# Supplementary material for: Kojic Acid Dipalmitate-Loaded Nanoparticles for the Treatment of Triple-Negative Breast Cancer
Source: J Nanotechnol Nanomater. Author manuscript; Available in PMC 2026 Jul 21. (PMC13384227)
Supplement: Supplementary Material [file NIHMS2186078-supplement-Supplementary_Material.pdf]

__Citation:__ Zilles JC, Obidiro O, Battogtokh G, Zimmer AR, Contri RV, Akala EO. Kojic Acid Dipalmitate-Loaded Nanoparticles for the Treatment Triple-Negative Breast Cancer. J Nanotechnol Nanomaterials. 2026;7(1):1–17.

Supplementary Material

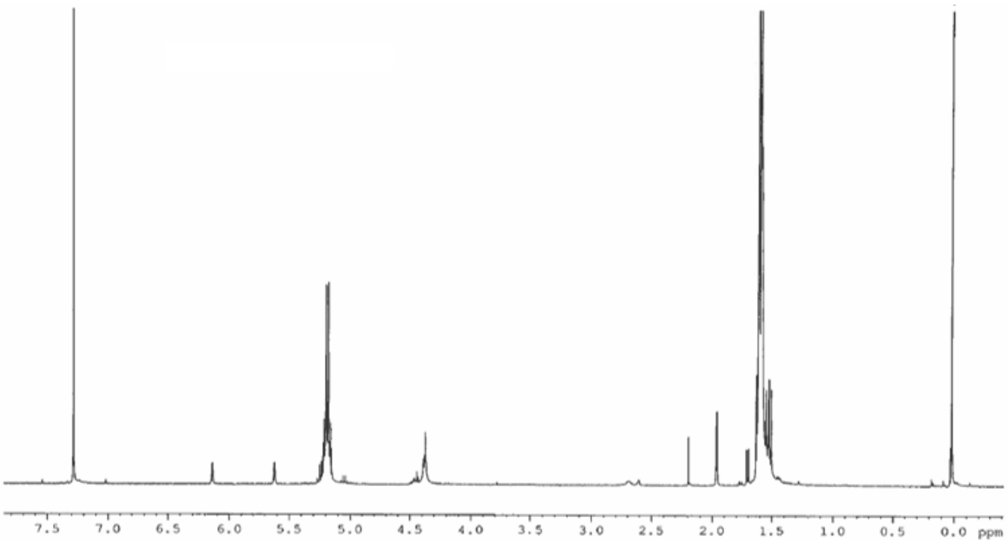

Supplementary Figure 1. ^{1}H-NMR spectrum of the macromonomer.

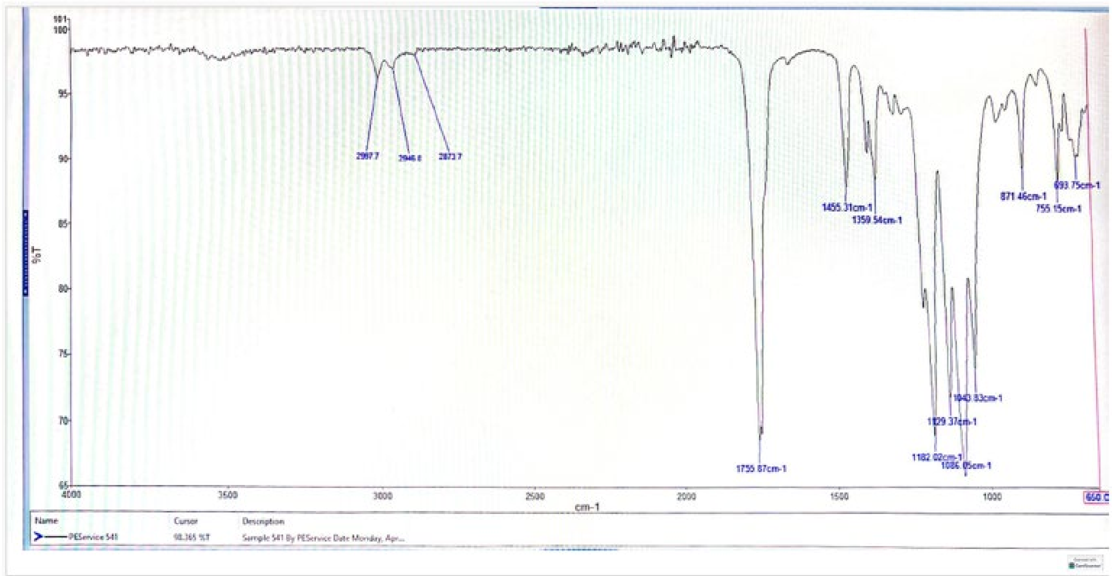

Supplementary Figure 2. FTIR spectrum of the macromonomer.

***Citation:** Zilles JC, Obidiro O, Battogtokh G, Zimmer AR, Contri RV, Akala EO. Kojic Acid Dipalmitate-Loaded Nanoparticles for the Treatment Triple-Negative Breast Cancer. J Nanotechnol Nanomaterials. 2026;7(1):1–17.*

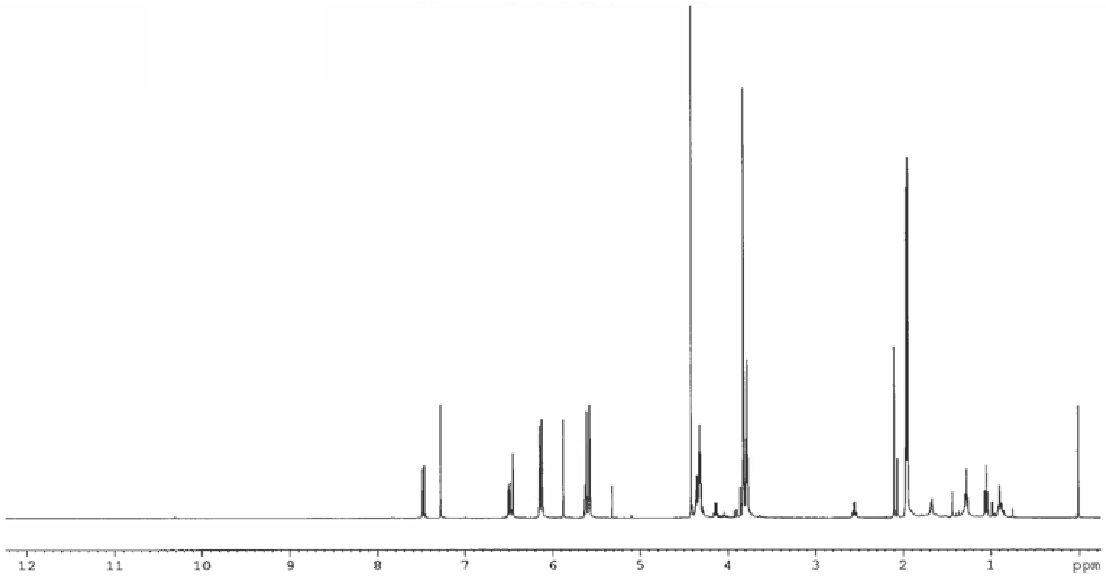

Supplementary Figure 3. ^{1}H-NMR spectrum of the purified crosslinker.

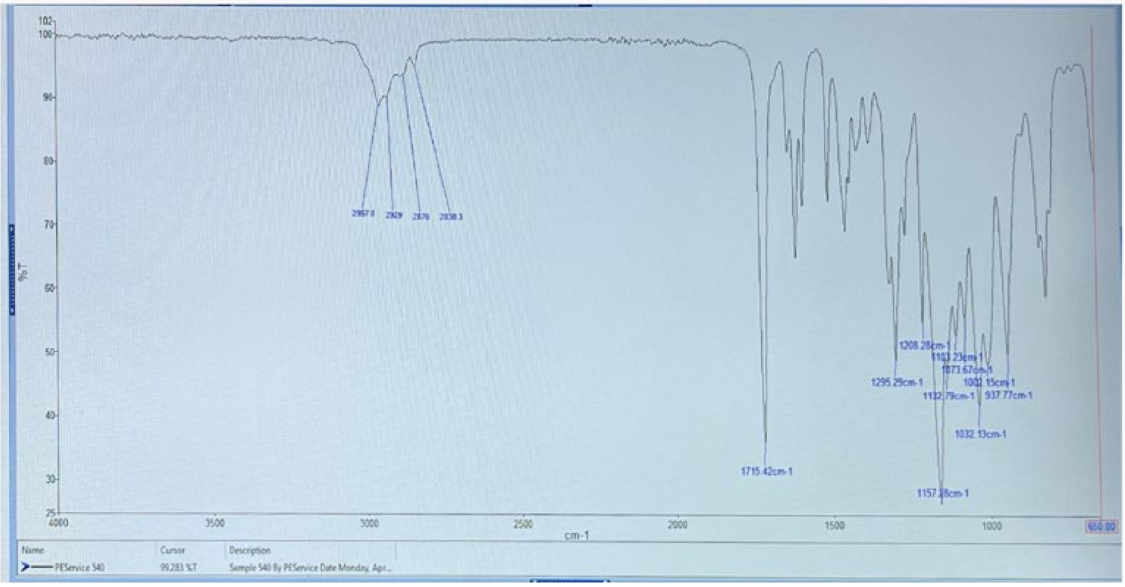

Supplementary Figure 4. FTIR spectrum of the purified crosslinker.

Citation: *Zilles JC, Obidiro O, Battogtokh G, Zimmer AR, Contri RV, Akala EO. Kojic Acid Dipalmitate-Loaded Nanoparticles for the Treatment Triple-Negative Breast Cancer. J Nanotechnol Nanomaterials. 2026;7(1):1–17.*

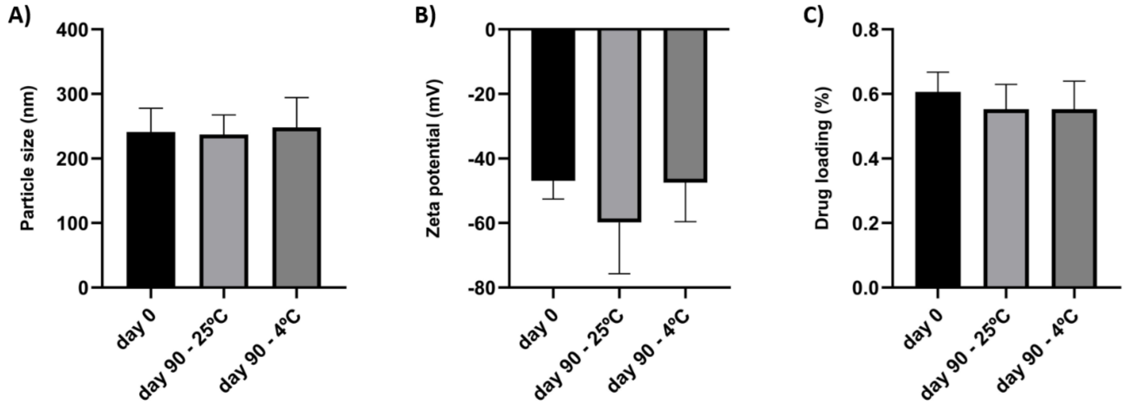

Supplementary Figure 5. Stability of nanoparticles at day 0 and after 90 days at room temperature storage (25°C) and refrigerated storage (4°C): particle size (**A**), zeta potential (**B**) and drug loading (**C**).
